# Otalgia from Reflex Irritation

**Published:** 1882-09

**Authors:** 


### OTALGIA FROM REFLEX DENTAL IRRITATION.

Dr. Bryson Delavan, M.D., of New York, contributes this article to the *American Journal of Otology*:

Otalgia, due to reflex irritation, is probably of more frequent occurrence than has heretofore been supposed. Not to mention the commonly-observed phenomenon of pain in the ear, accompanying acute affections of the tonsil, it is well known, also, as a not unusual and an extremely painful complication of tubercular cancerous, and syphilitic disease of the pharyngeal region. The writer has seen two cases where otalgia, due to malignant disease of the pharynx, was treated locally for a considerable length of time before the presence of the cancer was recognized.* Although it is not strange that irritation in other parts of the buccal cavity, equally remote from the ear, should result in the same reflex neuralgic symptoms, it is remarkable that they should occur upon the side *opposite* to the point of irritation. As illustrating the possibility of this, however, the following case possesses interest.

The patient was a finely developed, well nourished girl of about twenty, with clear complexion, active circulation and every apparent indication of robust health. Her family history was excellent, and there was no suggestion of any heredity. She had always enjoyed perfect health, with the exception of an attack of double otitis media, at age of seven. At this time there was a purulent discharge from both ears. Since then has always been slightly deaf in the right ear. The present attack began with a pain in the right ear. The pain was constant and throbbing, with occasional paroxysms of lancination, and was much more severe at night. This condition grew worse, producing almost complete insomnia. She was seen four days after the beginning of the attack. Examination with the otoscope revealed both tympani in a healthy condition, with no sign whatever of inflammatory action either of the external or of the middle ear. Rhinoscopic examination failed to discover any cause in the pharynx, both the tonsils and the parts in the retro-nasal space being remarkably free from hyperæmia. The upper teeth were perfect. In the lower jaw the right second molar was slightly carious, while the right wisdom tooth had not yet made its appearance. On the left side the wisdom tooth was through, but the second molar was wanting. Patient stated that several years

* "Primary Epithelioma of Tonsil."—*New York Medical Journal, April*, 1882.

ago this latter tooth had decayed and come out. Application of heat and anodynes to the affected ear gave temporary relief, but their good effects never lasted more than three or four hours. The otalgia was apparently reflex, and seemed due, in all probability, to irritation from the developing wisdom tooth of the right side, or from the carious second molar of the same side. The patient was seen in consultation by Dr. Samuel Sexton, who agreed with this hypothesis. At this suggestion she was referred to a competent dentist, who discovered that the deficient second molar of the left side had only lost its crown, while the roots were still impacted in the jaw, although completely covered by mucous membrane. With considerable difficulty three large roots were removed. At the extremity of one of these a sacculated abscess of extraordinary size was found. The wound caused by the removal of these fragments soon healed, and the otalgia quickly disappeared. Since the operation--two months--the carious right second molar has not been filled, nor has the right wisdom tooth yet completed its irruption. Nevertheless, there has been no return of the otalgia whatever.

Few instances, if any, have been reported where irritation, not only far removed, but actually upon the side opposite to the neuralgic manifestation, has resulted as in the case above. As yet, from the almost immediate relief which followed the operation, there can be no reasonable doubt as to the relation of cause and effect between the alveolar abscess and the neuralgia.

It appears, therefore, that a disordered condition of the teeth may exert a powerful influence upon parts with which they seem to have but little nervous connection, and the inference is forced upon us that in many cases of cephalic neuralgia the true cause lies in dental irritation, so that relief is not likely to be afforded until the irritation has been removed.—*Exchange.*

